# Bovine Neonatal Pancytopenia-Associated Alloantibodies Recognize Individual Bovine Leukocyte Antigen 1 Alleles

**DOI:** 10.3389/fimmu.2018.01902

**Published:** 2018-08-14

**Authors:** Rahel Kasonta, Jacqueline Mauritz, Christina Spohr, Carola Sauter-Louis, Karin Duchow, Klaus Cussler, Mark Holsteg, Max Bastian

**Affiliations:** ^1^Paul-Ehrlich-Institut, Langen, Germany; ^2^Friedrich-Loeffler-Institut, Greifswald-Insel Riems, Germany; ^3^Landwirtschaftskammer Nordrhein-Westfalen, Bonn, Germany

**Keywords:** veterinary vaccines, bovine neonatal pancytopenia, bovine leukocyte antigen I, alloantibodies, alloimmunity

## Abstract

Bovine neonatal pancytopenia (BNP) was a vaccine-induced alloimmune disease observed in young calves and characterized by hemorrhages, pancytopenia, and severe destruction of the hematopoietic tissues. BNP was induced by alloreactive maternal antibodies present in the colostrum of certain cows vaccinated with a highly adjuvanted vaccine against bovine viral diarrhea. Bioprocess impurities, originating from the production cell line of the vaccine, are likely to have induced these alloreactive antibodies. One prominent alloantigen recognized by vaccine-induced alloantibodies is highly polymorphic bovine major histocompatibility complex class I antigen (bovine leukocyte antigen 1—BoLA I). Aim of this study was to define the fine specificity of BNP-associated anti-BoLA I alloantibodies. In total, eight different BoLA I alleles from the production cell line were identified. All genes were cloned and recombinantly expressed in murine cell lines. Using these cells in a flow cytometric assay, the presence of BoLA I specific alloantibodies in BNP dam sera was proven. Three BoLA I variants were identified that accounted for the majority of vaccine-induced BoLA I reactivity. By comparing the sequence of immunogenic to non-immunogenic BoLA I variants probable minimal epitopes on BoLA I were identified. In general, dams of BNP calves displayed high levels of BoLA I reactive alloantibodies, while vaccinated cows delivering healthy calves had significantly lower alloantibody titers. We identified a subgroup of vaccinated cows with healthy calves displaying very high alloantibody titers. Between these cows and BNP dams no principle difference in the BoLA I reactivity pattern was observed. However, with a limited set of dam-calf pairs it could be demonstrated that serum from these cows did not bind to BoLA I expressing leukocytes of their offspring. By contrast, when testing cells from surviving BNP calves with the corresponding dam’s serum there was significant binding. We therefore conclude that predominantly highly alloreactive cows are at risk to induce BNP and it depends on the paternally inherited BoLA I whether or not the calf develops BNP.

## Introduction

Bovine neonatal pancytopenia (BNP) was a hemorrhagic disease in young cattle that emerged in most EU states in 2007 (1–5) and later in New Zealand (6). The condition was characterized by an increased susceptibility to bleeding from various body parts in young calves and a high-mortality rate. The cause of internal and/or external bleeding was a widespread and severe destruction of the bone marrow, resulting in depletion of hematopoietic stem cells leading to thrombocytopenia, non-regenerative anemia, and leukopenia (3, 7). Epidemiological data and experimental studies revealed that the dams that gave birth to calves affected by BNP were immunized with PregSure^®^BVD, a highly adjuvanted vaccine against bovine viral diarrhea (BVD) (4, 8). Several publications thereafter showed that bioprocess impurities in the vaccine, originating from the cell line used for viral propagation and vaccine production induced these alloreactive antibodies in the vaccinated cattle (1, 9). BNP-associated alloantibodies target highly polymorphic major histocompatibility complex class I antigen (MHC-I) (bovine leukocyte antigen 1—BoLA I) (10, 11).

The proposed BNP model hypothesizes that the cell line-derived BoLA I variants are found as bioprocess impurities in the vaccine (10, 11). Upon vaccination, a cow mounts an antibody response against those variants that are foreign to her. If the calf inherits one of these immunogenic variants from the father, the BNP alloantibodies from the dam can bind and then lead to disease induction in the calf. In this study, we aimed to investigate the fine specificity of anti-BoLA I alloantibodies in more detail. To this end, we established a eukaryotic expression system with murine cell lines expressing individual BoLA I alleles derived from the production cell line. The transduced cell lines were tested by flow cytometry using sera from PregSure^®^BVD vaccinated cows.

## Materials and Methods

### Field Cases and Clinical Material

The majority of BNP cases in this study were identified by Mark Holsteg of the Rindergesundheitsdienst (Cattle Health Service), North Rhine Westfalia. BNP diagnosis based on clinical findings was confirmed by hematology and bone marrow biopsy or post-mortem. All cases have been reported to and reviewed by the national pharmacovigilance system. The vast majority of sera were obtained by venipuncture for BNP-unrelated, diagnostic purposes and was stored frozen until transferred for further investigation to the Paul-Ehrlich-Institut. In total, 969 animals were available for analysis. Control sera from 21 animals were sampled from animals that had not been vaccinated against BVD. In total, 851 animals had been vaccinated with PregSure^®^BVD but had delivered healthy calves (hereafter referred to as non-BNP dams). 97 dams gave birth to bleeder calves, i.e., were confirmed BNP dams. All BNP dams had received at least two vaccinations with PregSure^®^BVD according to the recommended vaccination scheme and most of them had received annual booster vaccinations.

Furthermore, we obtained serum and whole blood for generation of lymphoblasts, from seven different calves and their respective dams. Out of the seven dams, three had given birth to calves affected by BNP in the previous years. The complete vaccination history of all animals included in this study is available to the authors. This part of the study was announced to and approved by the local competent authority (State Office for Nature, Environment and Consumer Protection of North-Rhine-Westfalia, LANUV Recklinghausen, Germany; ref. 84-02.05.40.14.032).

### Cell Preparation and Cell Culture

The bovine kidney cell line used to produce PregSure^®^BVD was kindly provided by Pfizer Animal Health. Continuous cell culture was performed in accordance to instructions provided by the manufacturer. The cell line was tested to be free of BVDV.

Bovine leukocytes were prepared from whole blood by ammonium-chloride lysis. Briefly, 20 ml unclotted blood was centrifuged for 10 min at 400 × *g*. The supernatant was removed. The pelleted erythrocytes were lysed for 10 min by the addition of a buffer containing 0.15 M NH_4_Cl, 10 mM NaHCO_3_, and 0.1 mM EDTA. Cells were again centrifuged, and the resulting leukocyte pellet was washed once with PBS and then additionally purified by Ficoll Paque (1.077 g/ml; GE Healthcare) gradient centrifugation: leukocytes were re-suspended in PBS containing 0.5% FCS, carefully layered on Ficoll Paque, and centrifuged for 20 min at 400 × *g* with low deceleration rate. The interphase was recovered and the resulting PBMC pellet was washed twice with PBS containing 0.5% FCS. For individual experiments, short-term T cell lines were obtained by phytohemagglutinin (PHA) stimulation. To this end, PBMCs were re-suspended in complete medium [i.e., RPMI 1640 (Gibco) supplemented with l-glutamine, penicillin–streptomycin, and 10% FCS] and stimulated at 1 × 10^6^ cells/ml with 0.1 µg/ml purified PHA (Oxoid) and a 1:20 dilution of a hybridoma supernatant containing human IL-2. The resulting polyclonal T cell lines are hereafter referred to as lymphoblasts.

### BoLA I Sequencing

Bovine leukocyte antigen 1 alleles expressed on the PregSure^®^BVD production cell line, were identified by reverse transcription polymerase chain reaction (RT-PCR), using the conventional methods. Briefly, the mRNA was extracted from the bovine kidney cell line, using an mRNA extraction kit (RNeasy Mini Kit, Qiagen). By RT-PCR with BoLA-specific primers (Fwd: 5′ GATCCATGGGGCCGCGAACC 3′, Rev: 5′ CTCGAGTCACCCTTTAGGAACCG 3′; Thermo Fisher Scientific), the extracted BoLA I mRNA was amplified and cloned into the pGEM-T easy cloning vector (Promega), as described in the instructions included. Individual clones were picked, plasmid DNA extracted, and sequenced (StarSeq Germany, Mainz). The obtained sequences were aligned to the IPD-MHC database hosted by the EMB-EBI homepage (https://www.ebi.ac.uk/ipd/mhc/group/BoLA).

### Expression of BoLA I Alleles

Recombinant cell lines expressing the identified BoLA I alleles were generated by integrating the eukaryotic expression vector *pMyc*-IRES-eGFP (kindly donated by Klaus Karjalainen; Nanyang Technological University) into the murine pre-B cell line 38B9, using a retroviral moloney-mouse-leukemia-virus (MoMLV) based transduction system (Mass. Institute of Technology). To this end, the BoLA I insert was ligated into the *pMyc*-IRES-eGFP vector using BamHI- and XhoI-restriction sites and transformed into One Shot^®^ TOP10 Chemically Competent *E. coli* (Invitrogen), following instructions provided. Colonies were screened by PCR, positive clones were isolated and the plasmid DNA isolated using the *NucleoBond Xtra Midi-Kit* (Machery-Nagel).

5 × 10^6^ Plat-E packaging cells (Cell Biolabs Inc.), stably expressing MoMLV-gag-pol and env, were transfected with plasmid by CaCl_2_ transfection, as described (12). After approximately 15 h, the medium was exchanged with fresh SF-IMDM containing 10% FCS, and after 24 h the supernatant containing virus particles was harvested. Cell debris was removed by centrifugation at 3,000 × *g* for 10 min at 4°C, followed by another centrifugation step at 10,000 × *g* for 1 h at 4°C. The pelleted virus was added to 38B9 cells pre-cultured in 2 ml SF-IMDM media containing 2% FCS. As a measure of transduction efficiency GFP fluorescence was assessed after 24 h by fluorescence microscopy. Subsequently, transduced GFP-positive cells were subjected to limiting dilution to obtain homogeneous, GFP-fluorescent cells. Transduced and non-transduced cells were further analyzed by flow cytometry.

### Flow Cytometry

To examine samples for the presence of opsonizing alloimmune antibodies, flow cytometry analyses were carried out as previously described (13). Briefly, up to 1 × 10^5^ cells of the production cell line or lymphoblasts were re-suspended in PBS containing 0.5% fetal calf serum (FCS, Gibco). Bovine sera were added to a final dilution of 1:5 (if not stated otherwise) followed by 1 h incubation at 4°C. Cells were then washed twice with PBS containing 0.5% FCS and cell surface bound bovine IgG detected using a FITC-conjugated polyclonal sheep-α-bovine IgG antibody (Invitrogen). Median fluorescence intensity (MFI) of living cells as defined by forward-scatter/sideward-scatter gating was determined for each sample using a BD Accuri C6 Flow Cytometer.

BoLA-I expression on transduced cell lines was analyzed by flow cytometry using the mouse anti-BoLA I monoclonal antibody H58A (VMRD). To directly assess background binding unrelated to the transduction, transduced and non-transduced 38B9 cells were mixed in a 1:1 ratio and treated with a 1:50 serum dilution. Binding was detected with a biotinylated anti-bovine antibody (AbD Serotec) and streptavidin coupled to PE-Cy5.5 (Invitrogen) in PBS with 0.5% FCS, thus reactive samples are GFP positive as well as PE-Cy5.5 positive. Fluorescence overlap was compensated by subtracting 0.6% of FL1.

### Statistical Analysis

The MFI was determined by the CFlowPlusAnalysis Software (BD). The results are presented as the quotient of the MFI (of transduced 38B9 cells)/MFI (non-transduced 38B9 cells) to exclude background staining to non-transduced cells. Experiments were performed in multiples of at least three independent replicates and of these representative data are presented. When applicable, the *p*-value (significance level α = 0.05) was calculated by non-parametric Mann–Whitney test.

## Results

### PregSure^®^BVD Immunized Animals React to the BK Cell Line

**Figure 1:**
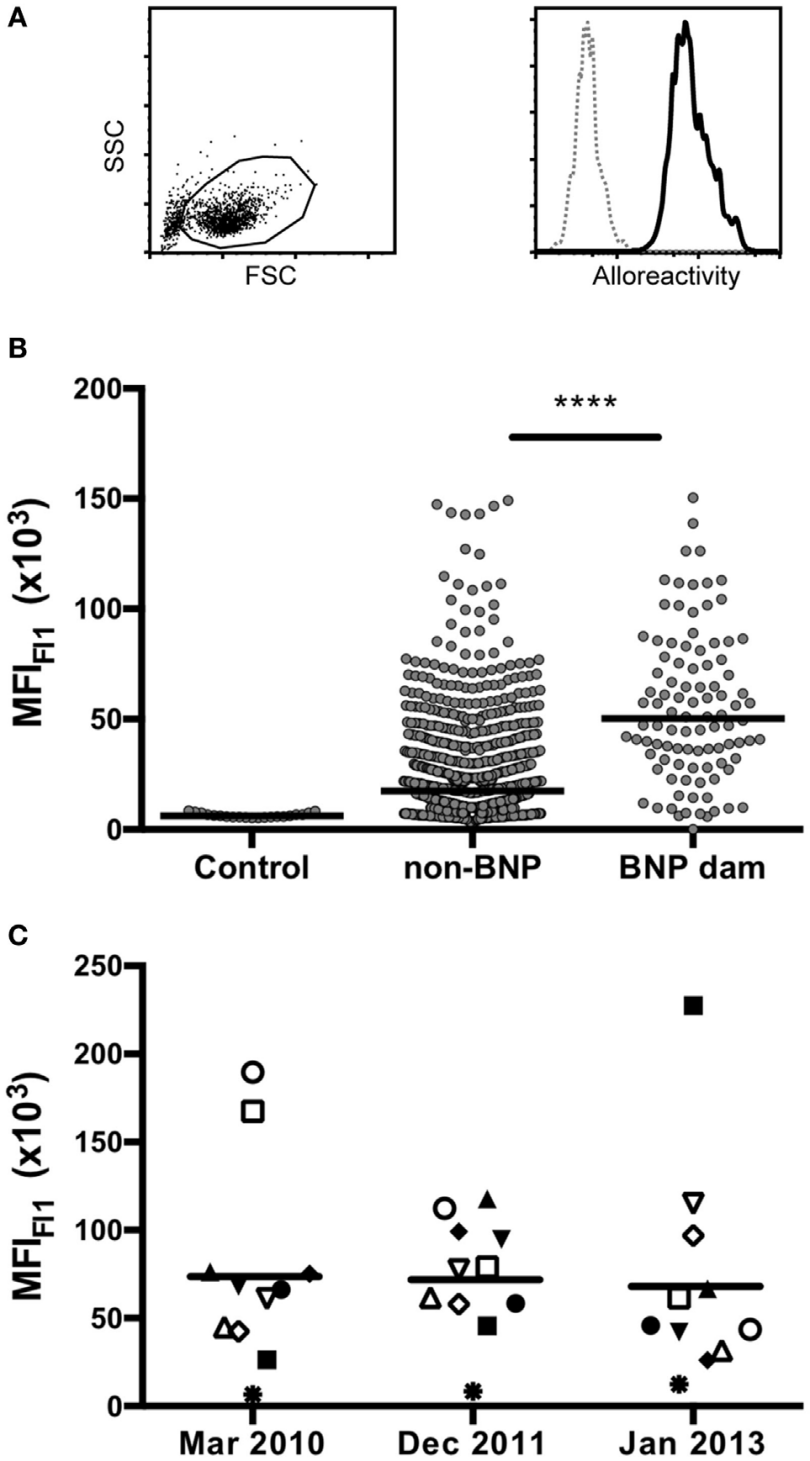
Flow cytometric reactivity to the PregSure^®^BVD production cell line. **(A)** Cells of the PregSure^®^BVD production cell line were investigated by flow cytometry. The left panel shows the forward-/side-scatter characteristics. The right panel represents a histogram showing the fluorescent labeling after coincubation with serum of a bovine neonatal pancytopenia (BNP) dam (bold line) or a non-vaccinated control (dotted line). **(B)** Alloantibody binding was determined for 21 control sera from non-vaccinated animals (control), 854 sera from PregSure^®^BVD vaccinated animals that gave birth to healthy calves (non-BNP), and 98 sera from BNP dams (BNP dam). Non-parametric Mann–Whitney test was used to determine the significance of differences. Four asterisks indicate a *p*-value < 0.0001. **(C)** From 11 BNP dams sera were obtained over three consecutive years and tested for alloreactive binding. Individual symbols represent the mean fluorescence at the respective time point. Black bars represent the group mean.

**Figure 2:**
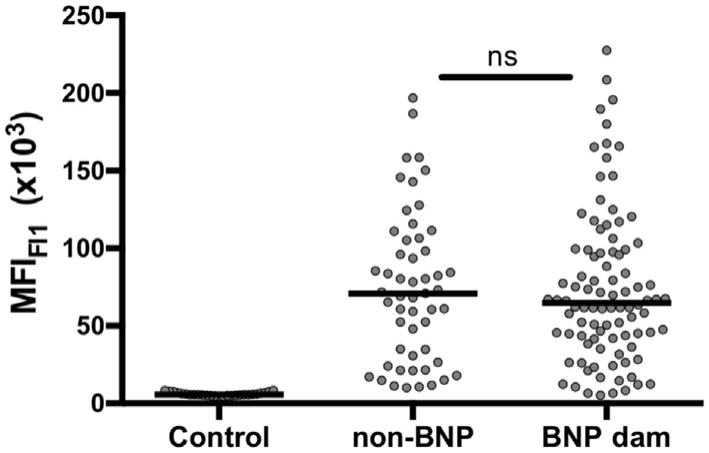
Selecting a serum panel of high-reacting non-bovine neonatal pancytopenia (BNP) dams. A panel of 53 sera from high-reacting non-BNP dams (non-BNP) was selected and alloantibody binding was compared with 21 control sera and 98 BNP dam sera (BNP dam). By non-parametric Mann–Whitney test, it was confirmed that no significant difference existed between the selected panel of high-reacting non-BNP dams and the BNP dam sera.

A comprehensive panel of 969 serum samples that had been collected over the course of 3 years by Mark Holsteg was analyzed for alloreactive binding to the BK cell line that had been used for the production of PregSure^®^BVD using a previously described flow cytometric assay (13). The data presented in Figure 1 confirm previous findings, as BNP dams showed a significantly higher BNP-associated alloantibody titer compared with control animals or PregSure-immunized non-BNP dams (Figure 1B). From 11 animals, we obtained three consecutive serum samples. It is of note that there was no general decline of alloantibody titers although the last PregSure^®^BVD immunization has been administered in that herd by the end of 2009 (Figure 1C). From the entire panel of non-BNP dam, sera 53 were selected that displayed a strong alloreactivity although the respective cows had delivered healthy calves. Between these high-reacting non-BNP dams and the BNP mothers, there was no statistical difference in the alloantibody binding (Figure 2). This preselected panel of sera was used for the following analyses.

### Recombinantly Produced Murine Cell Lines Express BoLA Class I

**Figure 3:**
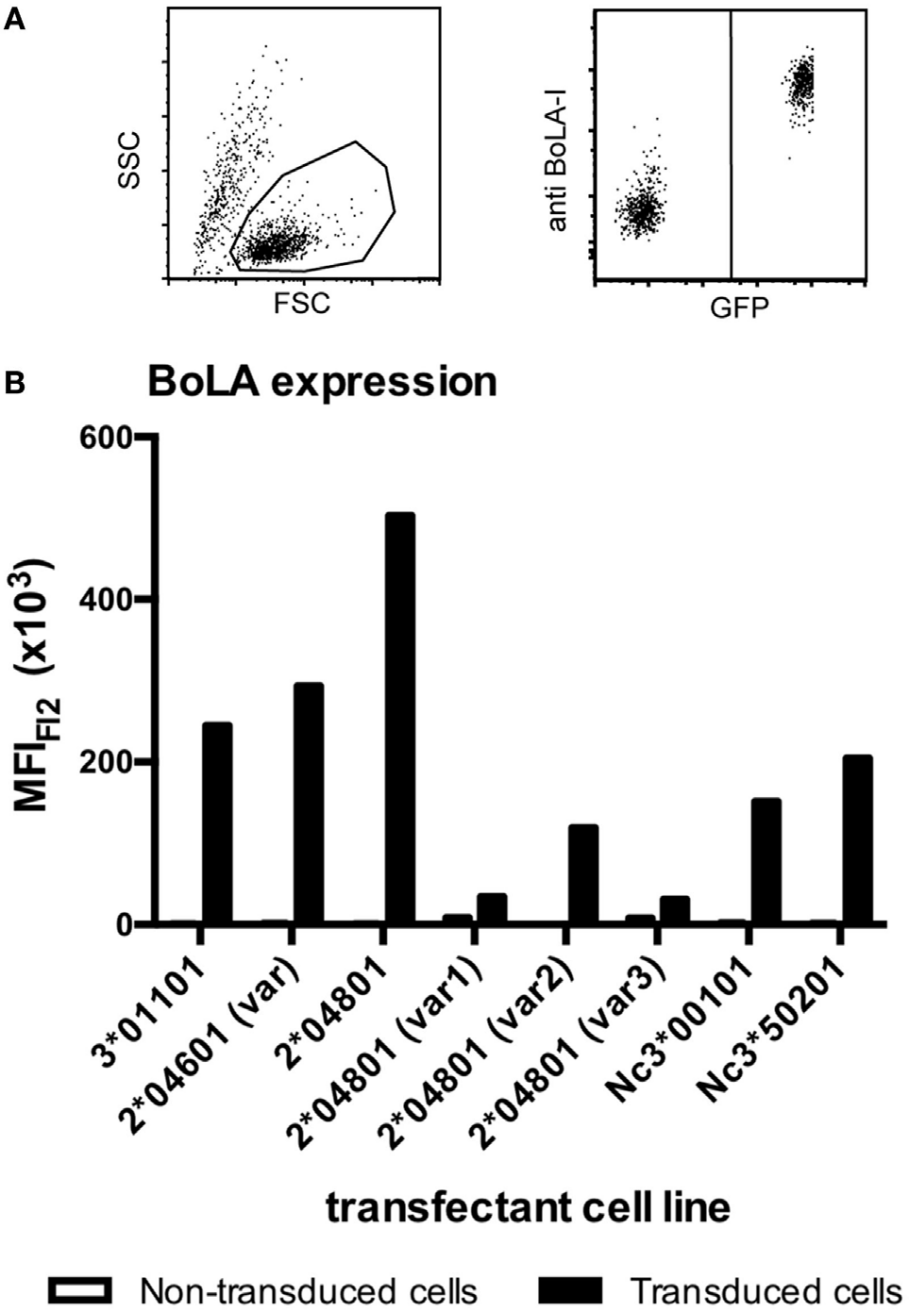
Transduced murine cell lines express bovine leukocyte antigen 1 (BoLA I). BoLA I genes were cloned and stably expressed in a murine pre-B cell line using a moloney-mouse-leukemia-virus-based transduction system. Transduced cells expressed the BoLA I allele of interest together with GFP. To test for the expression of BoLA I, transduced and non-transduced cells were coincubated with a monoclonal anti-BoLA I antibody. Antibody binding was revealed using an anti-murine PE-Cy5.5 conjugated secondary antibody. Subsequently, cells were analyzed by flow cytometry. **(A)** The left panel shows the gating strategy according to forward-/side scatter characteristics. The right panel shows GFP- and BoLA I expression: non-transduced cells are GFP and PE-Cy5.5 negative (bottom left corner), transduced cells are GFP- and BoLA I positive (upper right corner). **(B)** The respective transduced cell lines were analyzed for the expression of BoLA I. Black bars represent the mean fluorescence after BoLA I staining of GFP-positive, transduced cells. Open bars represent GFP-negative, non-transduced cell lines.

Bovine leukocyte antigen 1 genes of the PregSure^®^BVD production cell line were isolated and amplified by RT-PCR using BoLA I specific primers. Sanger sequencing of cloned isolates identified in total eight productive and two non-productive BoLA I alleles. With one exception the eight productive sequences were identical to published BoLA I alleles or showed single non-synonymous nucleotide polymorphisms in the highly polymorphic alpha 1 and 2 region. Of two alleles several cDNA clones were identified. Of the other alleles only single clones were found (see Table 1). The eight productive BoLA I genes were cloned into the eukaryotic expression vector *pMyc*-IRES-eGFP and packaged into replication deficient MoMLV particles. These particles were used to transduce the murine Pre-B cell line, 38B9. Green GFP fluorescence was employed to monitor transduction efficiency using fluorescence microscopy. Expression of the different BoLA I isotypes on the cell surface was assessed by flow cytometry after staining with a monoclonal anti-BoLA I antibody. Expression of all BoLA I isotypes could be demonstrated (Figure 3). However, two alleles, BoLA 2*40801 (var 1) and BoLA 2*40801 (var 3), were expressed at a considerably lower level compared with the others.

**Table 1:** BoLA alleles sequenced from the PregSure^®^BVD production cell line.

Bovine leukocyte antigen 1 allele	*N*	Remarks
BoLA 3*01101	4	Full productive sequence, 100% match
BoLA 2*04801	7	Full productive sequence, 100% match
BoLA 2*04801 variant 1	1	Full productive sequence, 6 mismatches to BoLA 2*04801
BoLA 2*04801 variant 2	1	Full productive sequence, 29 mismatches to BoLA 2*04801
BoLA 2*04801 variant 3	1	Full productive sequence, 138 bp missing
BoLA 2*040601 variant	1	Full productive sequence, 35 mismatches to BoLA 2*04601
BoLA Nc3*50201	1	Full productive sequence, 100% match
BoLA Nc3*00101	1	Full productive sequence, terminates two amino acids too early
Similar to Nc2*00202	1	Non-productive sequence
Similar to Nc1*50002	1	Non-productive sequence

*N depicts the absolute number of cDNA clones identified*.

### Three BoLA I Alleles Are Recognized by PregSure^®^BVD Immunized Animals

**Figure 4:**
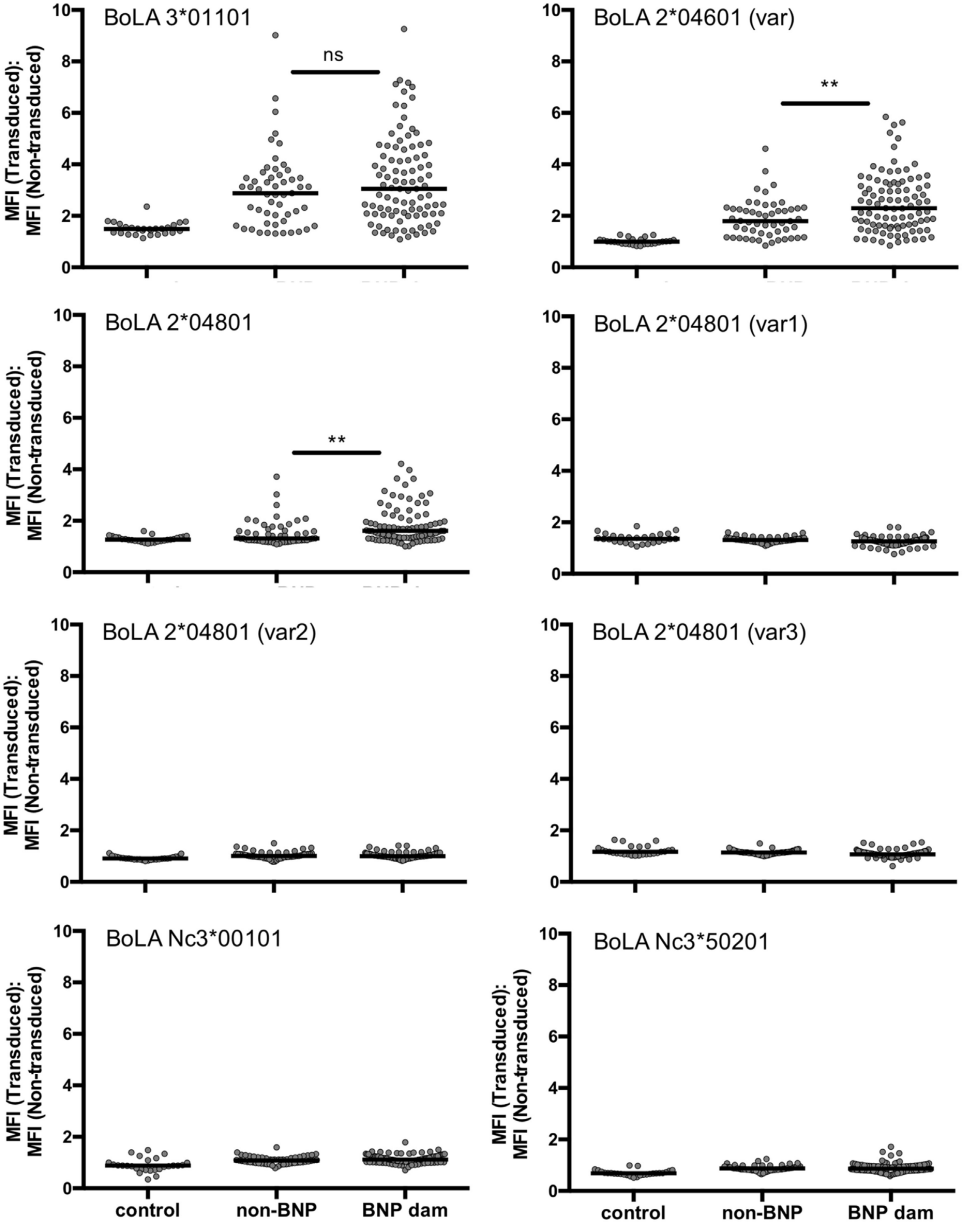
Bovine alloimmune sera recognize recombinantly expressed bovine leukocyte antigen 1 (BoLA I). Non-transduced and transduced cells expressing different BoLA I variants were coincubated with the same serum panels shown in Figure 2 [21 control sera; 53 high-reacting non-bovine neonatal pancytopenia (BNP) sera; 98 BNP sera]. Antibody binding was revealed using an anti-murine PE-Cy5.5 conjugated secondary antibody. Each graph stands for one cell line expressing the respective BoLA I variant. Gray circles represent the BoLA I specific binding of the tested serum depicted as ratio between mean fluorescence of transduced and non-transduced cells. Black bars represent the group median. Non-parametric Mann–Whitney test was used to determine the significance of differences. Two asterisks represent a *p*-value < 0.01.

One common hypothesis is that BoLA I specific alloantibodies cause BNP. To test this hypothesis we investigated, whether animals that reacted strongly to the BK cell line but gave birth to healthy calves (high-reacting non-BNP dams) recognize different BoLA I alleles than dams that gave birth to calves affected by BNP (BNP dam). To this end, the panel of preselected sera shown in Figure 2 was tested for BoLA-I specific binding using the recombinant BoLA I expressing cell lines. As we had previously observed xenoreactive binding of bovine sera to non-transduced, murine Pre-B cells, for the assay transduced and non-transduced cells were mixed. Specific binding to recombinantly expressed BoLA I was then determined by forming the ratio between alloantibody binding to green-fluorescent, transduced and non-fluorescent, non-transduced cells. From the eight transduced cell lines, cells expressing the BoLA alleles BoLA 3*01101, BoLA 2*04601 (var), and 2*04801 were frequently recognized by non-BNP dams and BNP dams, not by the controls (Figure 4). However, no principal difference between high-reacting non-BNP and BNP dams could be revealed. However, in Figure S1 in Supplementary Material, the reactivity to individual BoLA alleles is compared between BNP and non-BNP dam sera that bound at least one allele. In this comparison, non-BNP dam sera displayed a slightly higher reactivity to allele 3*01101. By contrast, the response to the other alleles, i.e., 2*04601 var and 2*04801, was significantly lower (see Figure S1 in Supplementary Material).

### Sequence Comparison of the Three Recognized BoLA I Alleles

**Figure 5:**
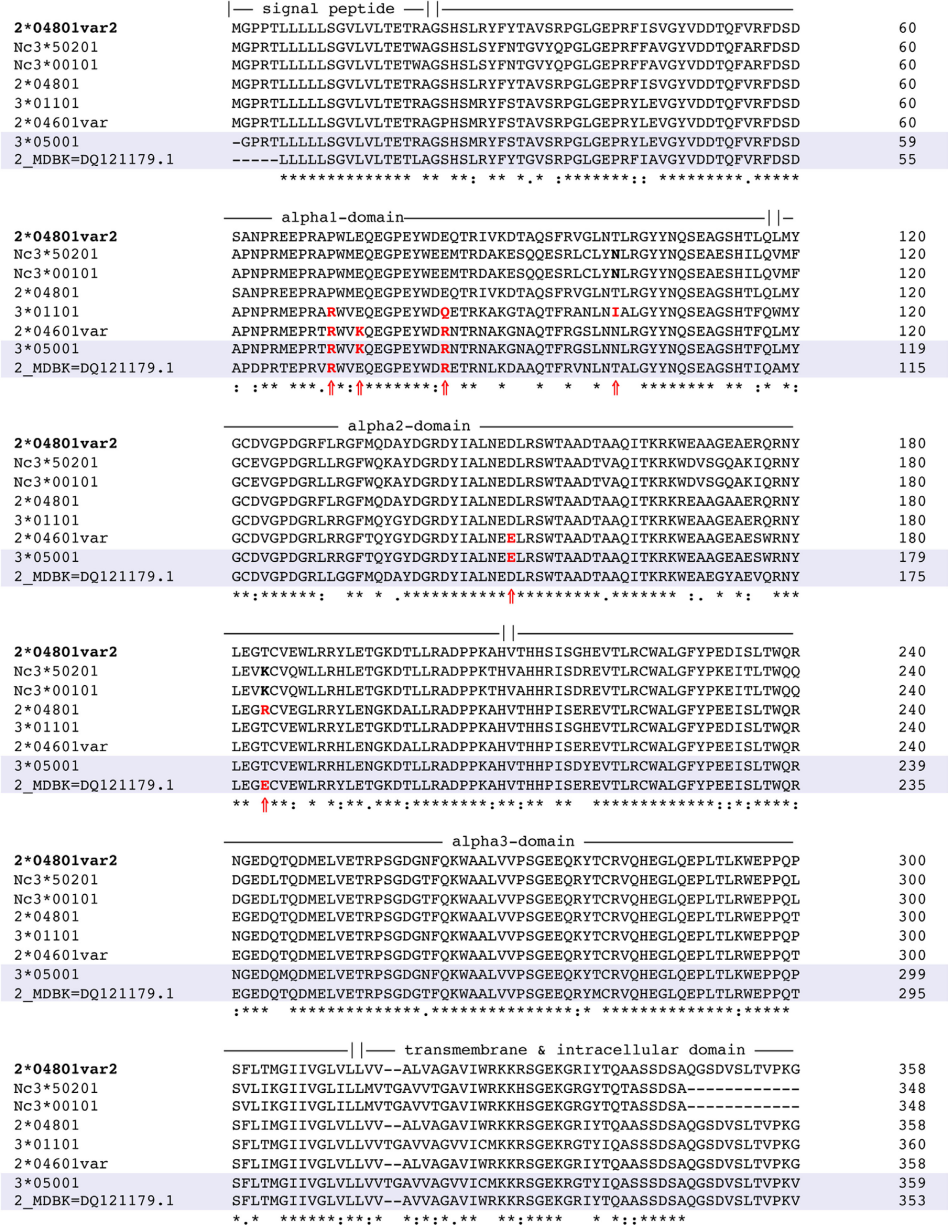
Multisequence alignment of recognized BoLA alleles recognized. The protein sequences of the three immunogenic bovine leukocyte antigen 1 (BoLA I) variants 3*01101, 2*04601, and 2*04801 were aligned to a variant that was expressed, but not recognized by bovine neonatal pancytopenia (BNP) sera [BoLA 2*04801 (var 2)]. The signal peptide, the alpha-1, alpha-2, alpha-3 domain, and the transmembrane region are indicated according to information available on the UniProt database. An asterisk (*) denotes that residues at that position are exactly the same in all sequences. A colon (:) indicates a conserved substitution, i.e., the residues share the same properties. A dot (.) indicates semi-conserved substitutions and a blank denotes that there is no common property. Amino acids marked in bold and with a black arrow (?) indicate positions that differ from the reference sequence and presumably protrude from the molecule. The numbers to the far right signify the position number of the nucleotide strand.

**Figure 6:**
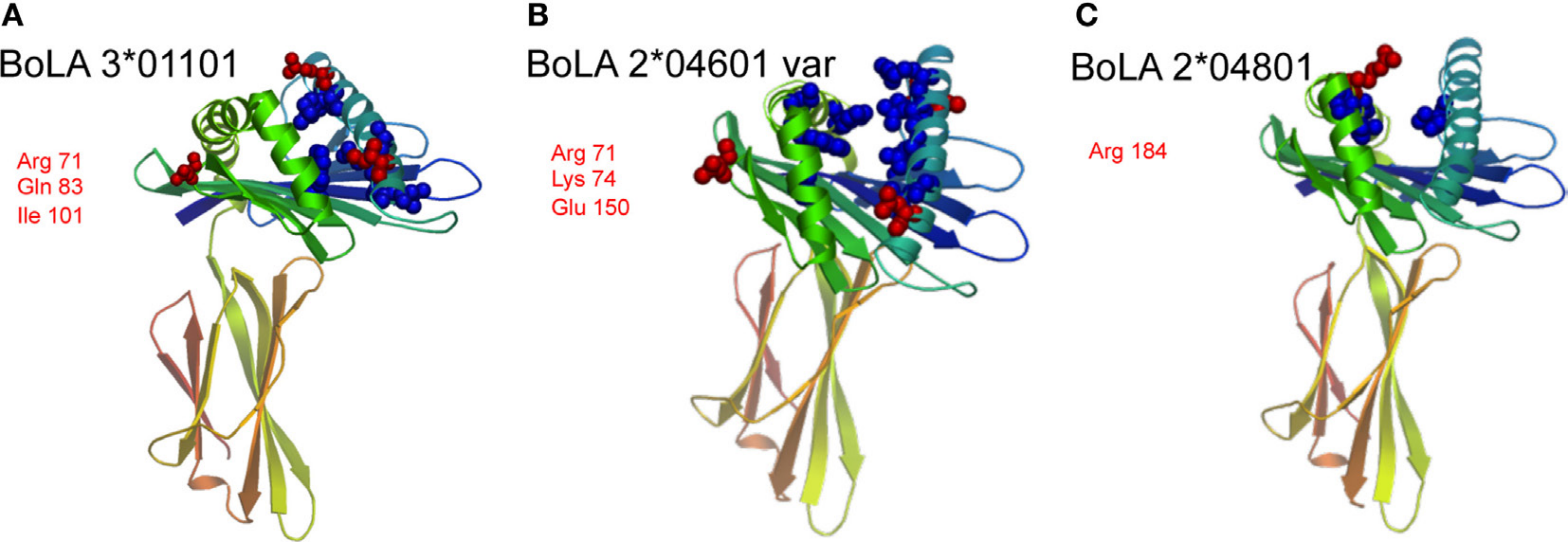
Protein modeling of the three recognized bovine leukocyte antigen 1 (BoLA I) variants. Using the SWISS-MODEL platform at http://www.proteinmodelportal.org, the three BoLA I recognized by alloimmune sera were modeled, panel **(A)** depicts BoLA 3*01101, panel **(B)** variant BoLA 2*04601 and panel **(C)** BoLA 2*04801. Those amino acids that differ from allele BoLA 2*04801 (var 2) and protrude from the molecule are depicted in red. Those that are buried inside are labeled in blue. The respective protruding amino acids and their positions are annotated using three letter code.

Overall, BoLA 3*01101 was most frequently recognized followed by BoLA 2*04601 (var) and BoLA 2*04801. A closely related variant of that allele [BoLA 2*04801 (var 2)] was not recognized at all. This allele was used as a non-recognized reference sequence. Protein-sequence alignment of these four BoLA I antigens allowed for deducing specific binding epitopes. Depicted in Figure 5 is the alignment of the three recognized BoLA antigens to the reference. Using the SWISS-MODEL platform (14–16) at http://www.proteinmodelportal.org the three BoLA alloantigens were modeled (Figure 6). Those amino acids that differ from allele BoLA 2*04801 (var 2) and protrude from the molecule are depicted in red. Those that are buried inside are labeled in blue. Notably, five of six potential antigenic amino acids contribute to the formation of the rim of the epitope binding groove.

### BNP Dams Recognize a Broader Spectrum of BoLA Alleles

**Figure 7:**
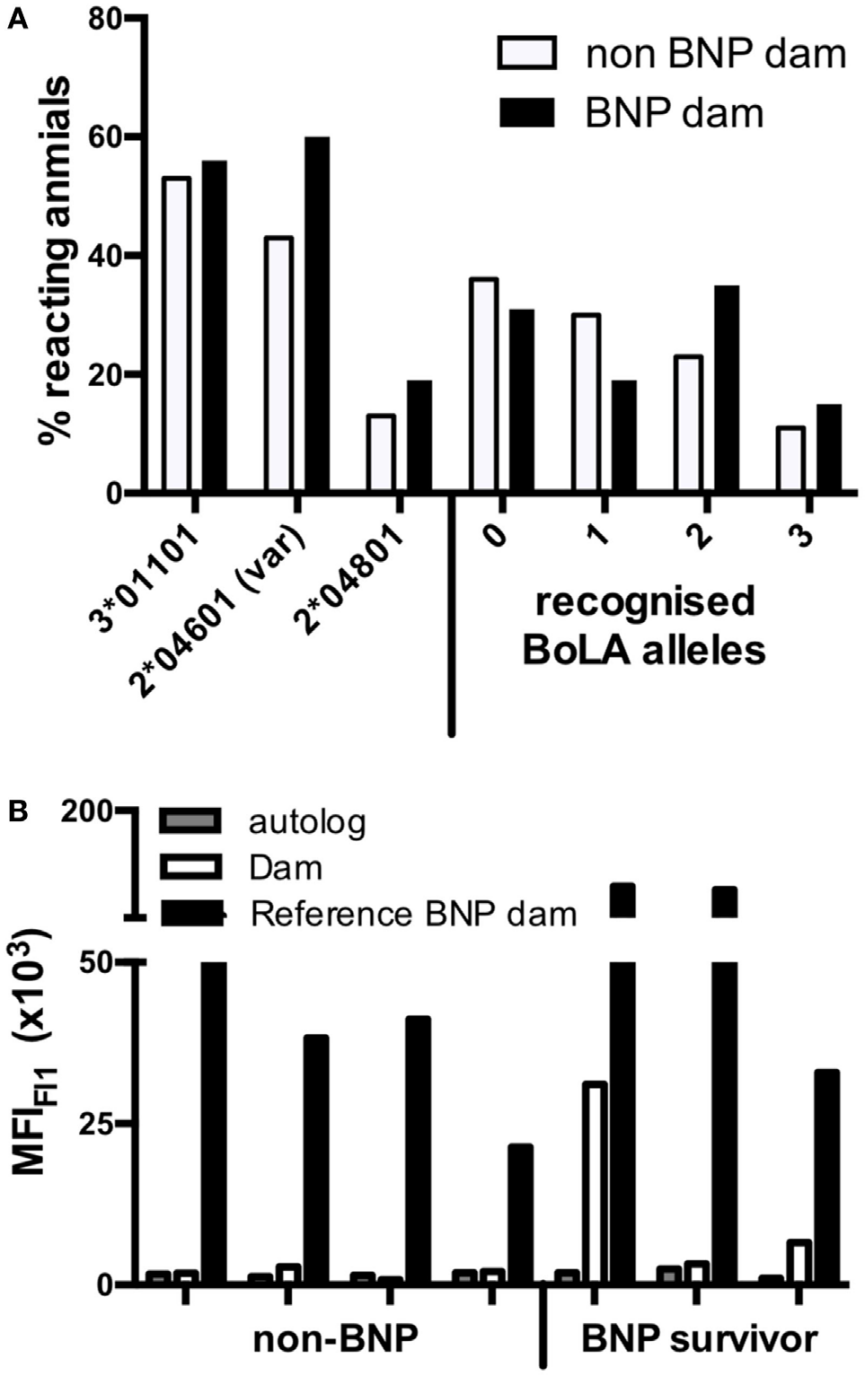
High-reacting non-bovine neonatal pancytopenia (BNP) dams recognize fewer bovine leukocyte antigen 1 (BoLA I) variants and do not bind to cells of their own calves. **(A)** The percentage of sera from high-reacting non-BNP dams (open bars) and BNP dams (black bars) that bound to the respective BoLA I variant is depicted on the left side. On the right side of the panel, the percentages of sera that reacted to none, one, two, or three different variants is shown. **(B)** Seven dam-calf combinations were investigated. PBMCs were obtained from four calves that were born by high-reacting, non-BNP dams and never showed any symptoms of BNP (non-BNP). In addition, PBMCs were obtained from three calves that survived an episode of BNP (BNP survivor). The alloantibody binding to the PBMCs was determined for autologous serum (gray bars), for the serum of the respective dams (open bars), and for the serum of an unrelated BNP-dam (black bars) and autologous sera (white bars). Bars represent the mean fluorescence as determined by flow cytometry.

There was no clear difference in the binding pattern between BNP dams and the preselected panel of highly reactive non-BNP dam sera. However, the percentage of animals that responded to BoLA I allele 2*04601 (var) was higher among BNP dams (Figure 7A, left). In addition, we found that BNP dams exhibited a broader reactivity: i.e., 55% of BNP dams recognized two or three alleles, whereas only 31% of non-BNP dams targeted 2 or more alleles (Figure 7A, right), making it more likely, that the maternal alloantibodies react with the calf’s paternally inherited BoLA alleles. To directly test this notion, we identified offspring of highly reactive non-BNP dams and progeny of BNP dams that survived an episode of BNP. Four adult cows from non-BNP dams and three adult BNP survivors were found in the herd. From these animals lymphoblast cell lines were established and tested in the flow cytometric binding assay. Binding of autologous serum was compared with that of the respective dam. As a reference one highly reactive BNP dam serum was tested in parallel. No reactivity whatsoever was seen with autologous sera or with non-BNP dam sera in combination with cells of their respective progeny with cells of their respective progeny, although all sera tested displayed a similar reactivity to the production cell line (see Figure S2 in Supplementary Material). By contrast, cells from two of the three BNP survivors were recognized by the serum of their respective dams. In one case, the reactivity was indistinguishable from background. The reference serum interacted with all cell lines (Figure 7B).

## Discussion

PregSure^®^BVD vaccinated cows harbor alloantibodies binding to the cell surface of the permanent bovine kidney cell line used for the manufacturing of PregSure^®^BVD. The induction of opsonizing alloantibodies was unique for this anti-BVDV-vaccine (1), which led to a voluntary sale stop in 2010 (17) and the withdrawal of the marketing authorization by the manufacturer. In line with a number of previous publications, we can confirm that BNP dams have significantly higher alloantibody titers compared with non-BNP dams, i.e., PregSure^®^BVD vaccinated cows that delivered healthy calves. Pioneering studies by F. Deutzkens and G. Foucras could demonstrate that PregSure^®^BVD induced, opsonizing alloantibodies bind BoLA I (2, 11). Due to the ubiquitous expression of BoLA I it used to be a matter of debate, how BoLA I targeting alloantibodies can induce the specific insult on haematopoetic stem cells observed in BNP affected calves. More recently, additional alloantigens, such as alpha3 and beta1 integrin were identified to be recognized by PregSure^®^BVD induced alloantibodies. However, it was also shown that the cell types that are most affected in BNP, for example, megakaryocytes, express particularly high levels of BoLA I while peripheral tissues, for example, the endothelium, only express low levels. In addition, it was observed that BoLA I specific alloantibodies contribute more to alloantibody binding of red bone marrow cells than integrin-specific antibodies (18). Probably, if high amounts of BoLA I specific antibodies are ingested with the colostrum, in peripheral tissues BoLA I molecules are rapidly saturated and the remaining antibodies reach and destroy the red bone marrow.

Among the 11 BNP dams, of which 3 consecutive serum samples over the course of 3 years were available to us, the mean alloantibody titer did not decline. This is of note because the last PregSure^®^BVD immunization in that herd had been administered 4 years before. Together, this indicates that the BoLA I specific alloreactivity is very long-lasting. This is in accordance with observations in humans (19) and may explain why there are still sporadic cases of BNP (Mark Holsteg, personal observation).

Using RT-PCR, we identified in total eight different BoLA I alleles, including two non-classical variants, from the PregSure^®^BVD production cell line. We cannot completely exclude the possibility that some of these single clones represent cloning artifacts. However, with the exception of one variant that lacked a stretch of 138 bp the remaining variants mainly showed single non-synonymous nucleotide polymorphisms in the highly polymorphic alpha 1 and 2 region and led to full length protein sequences. From that point of view, we consider it likely that the identified variants are truly expressed by the production cell line. In a similar approach, Benedictus et al. identified four classical BoLA I alleles in the MDBK cell line, which is held to be the production cell line (18). Two alleles were identical, but the presence of another two alleles could not be confirmed in our study. Instead, we observed another four variants that were not described previously. So far, six BoLA I loci have been mapped onto the chromosome 23, that means one individual can carry up to 12 different alleles (20, 21). As there may have been differences in the culture conditions, it could be that the discrepancy to the previous study is due to a potential down regulation or complete mRNA shutdown of individual BoLA I alleles. Alternatively, it is conceivable that there has been a “divergent evolution” of the MDBK cell line, i.e., potential mix-ups, during the long cultivation history before the master seed of the production cell line was established (22). The eight alleles that we were able to identify were recombinantly expressed in murine pre-B cell lines. Expression of BoLA I was demonstrated with monoclonal antibodies and by fluorescence microscopy (data not shown). With two variants, i.e., BoLA 2*40801 (var1) and BoLA 2*40801 (var3), only weak binding of the monoclonal anti-BoLA I antibody was observed. The respective recombinant cells were efficiently transduced, as seen by the bright GFP fluorescence (data not shown), and it was confirmed that the right coding sequences were integrated in the right order into the expression vector. However, it cannot be excluded that the monoclonal antibody bound weakly to these two BoLA I variants. This is particularly true for variant 3 of BoLA 2*40801 because in this sequence a stretch of 46 amino acids is missing.

Using the recombinant cell lines, we tested by flow cytometry which BoLA I antigens were recognized by BNP sera. The majority of PregSure^®^BVD induced alloantibodies was directed to the two variants BoLA 2*04601 (var) and BoLA 3*01101 and to a lesser extent to BoLA 2*04801. Therefore, we aligned these sequences to a closely related sequence that was not recognized at all [BoLA 2*04801 (var 2)]. Differences were mainly observed in the alpha-1 and alpha-2 domain. This is in accordance with the fact, that the first two domains form the epitope binding groove and represent the most polymorphic part of the molecule, while the alpha-3 domain interacts with the beta2-microglobulin and is less accessible to antibodies (23). Furthermore, computational modeling allowed us to identify differing amino acids protruding from the molecule. As depicted in Figure 6, we identified up to three amino acid exchanges per variant that could potentially form the minimal epitope of the vaccine-induced alloantibodies. This is in accordance with observations in human alloimmune conditions. Both, in neonatal alloimmune thrombocytopenia and post transfusion purpura single amino acid substitutions in the respective target proteins have been identified as epitopes of pathogenic alloantibodies (24–26). That none of the two identified non-classical BoLA I variants was recognized is in accordance with the fact that non-classical variants have a limited polymorphism and are thus less likely to represent alloantigens (27).

Although the majority of non-BNP dams shows significantly lower alloantibody titers compared with BNP mothers, we repeatedly identified animals with comparably high alloantibody binding. To test the hypothesis, whether these animals delivered healthy calves due to a different reactivity pattern to BoLA I antigens, we made a preselection of high-reacting non-BNP dams and compared their reactivity to that of BNP dams. It turned out that the three reactogenic BoLA I variants, namely, BoLA 3*01101, BoLA 2*04601 (var), and BoLA 2*04801, were frequently recognized both by BNP dams and non-BNP dams. Although there was a statistically significant difference in the reactivity against the latter two variants, this alone would probably not explain the difference in the clinical outcome.

However, the majority of non-BNP dams recognized none or only one of the recombinantly expressed BoLA I variants while the majority of BNP dams recognized two variants. This may indicate that non-BNP dams are less likely to have alloantibodies matching the BoLA I repertoire of their calves. We have previously described a pair of non-identical twin calves, one of which died due to BNP while the other showed no symptoms of BNP. In that study, we could demonstrate that the mother had no alloantibodies recognizing the BoLA I variants of the non-BNP sibling (28). Similarly, in this study, we could identify and analyze seven dam-calf combinations. With cells of two out of three heifers that anamnestically were reported to have survived a BNP episode as a calf, significant alloantibody binding with their dam’s serum was observed. By contrast, with none of the four non-bleeder calves alloantibody binding by their dam’s serum was detected. This was not due to a lack of BoLA I expression, since the cells were readily recognized by a BoLA I specific monoclonal antibody (data not shown). Also, when tested with BoLA I reactive serum from a non-related BNP dam, strong alloreactive binding was observed. So, the fact that high-reacting non-BNP dams also recognized recombinantly expressed BoLA I variants of the production cell line does not necessarily contradict the hypothesis that BoLA I is the causal alloantigen in the induction of BNP. The latter findings rather lead to the conclusion that their calves did not develop symptoms because they expressed BoLA I molecules not bound by the alloantibodies of their dams. This argues for a classical alloimmune pathogenesis where the alloantigen, i.e., the BoLA I allele, is paternally inherited. In the past, this notion, which was proposed early on (29), has partially been veiled by the fact that almost all PregSure^®^BVD vaccinated cows mount an alloimmune response to the BoLA I expressed by the production cell line (13). In addition, the finding that BNP is a heritable trait rather of the dam than of the sire (30) initially seemed to contradict the proposed model of a classical alloimmune phenomenon. However, the main implication of that observation is probably that the responsiveness to the vaccination is an intrinsic property of the dam. For the development of BNP this is decisive, because only if the dam has developed high levels of alloantibodies and passes them on to the calf *via* colostrum, they can overcome BoLA I expression in the periphery and reach the hematopoetic tissues. So far, the molecular and genetic background for that intrinsic property remains elusive. It is, however, well perceived that toll-like-receptor polymorphisms have a profound influence on human allograft rejection (31). Similarly, in the case of BNP, differences in innate pattern recognition receptors (PRRs) could govern the responsiveness to PregSure^®^BVD vaccination. As the field of innate receptors is ever expanding, it will be interesting to see which receptors are responding to the PregSure^®^BVD adjuvant and what polymorphisms they display.

## Ethics Statement

We obtained serum and whole blood for generation of lymphoblasts, from seven different calves and their respective dams. This part of the study was announced to and approved by the local competent authority (State Office for Nature, Environment and Consumer Protection of North-Rhine-Westfalia, LANUV Recklinghausen, Germany; ref. 84-02.05.2012.032).

## Author Contributions

RK, MH, KD, KC, and MB devised the study concept. MH identified BNP calves and contributed test sera. RK, JM, and CS performed the experimental work. CS-L helped with the statistical analysis. RK and MB wrote the manuscript. CS-L, KD, CS, KC, and MH helped to edit the manuscript.

## Conflict of Interest Statement

The authors declare that the research was conducted in the absence of any commercial or financial relationships that could be construed as a potential conflict of interest.

## Abbreviations

BNP, bovine neonatal pancytopenia; BoLA, bovine leukocyte antigen; BVD, bovine viral diarrhea; MFI, median fluorescence intensity; MHC-I, major histocompatibility complex class I antigen; NAIT, neonatal alloimmune thrombocytopenia; PEI, Paul-Ehrlich-institute; PRR, pattern recognition receptor.
