# Supplementary material for: Bovine Neonatal Pancytopenia-Associated Alloantibodies Recognize Individual Bovine Leukocyte Antigen 1 Alleles
Source: Front Immunol. 2018 Aug 14;9:1902. doi: 10.3389/fimmu.2018.01902 (PMC6102493; doi:10.3389/fimmu.2018.01902)
Supplement: Supplementary file 1 [file data_sheet_1.pdf]

Supplementary Figure S1

A

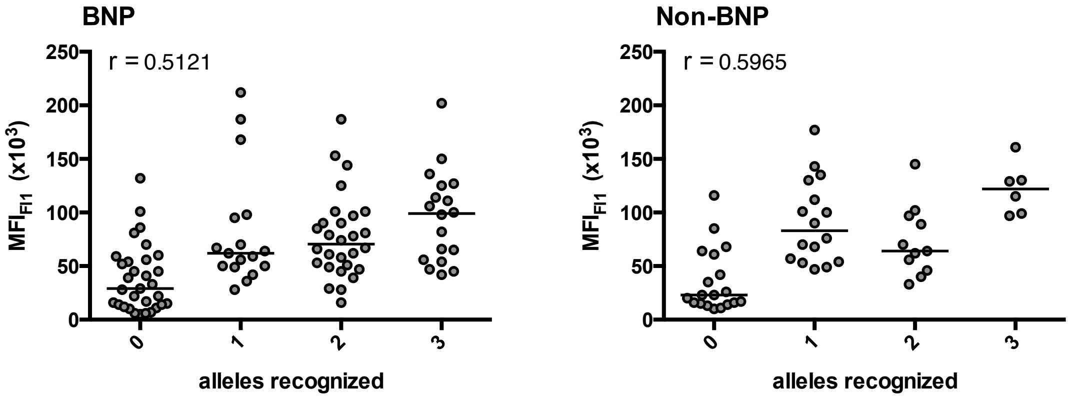

Reactivity to the production cell line was determined by flow cytometry and is compared for 94 BNP dam and 52 highly alloreactive, non-BNP dam sera. Sera are categorized according to the number of BoLA alleles recognized. Grey circles symbolize individual sera, black bars represent the group median. Correlation between alloreactivity and number of alleles recognized was calculated. Spearman's correlation coefficient r is depicted in the upper left corner of each graph.

B

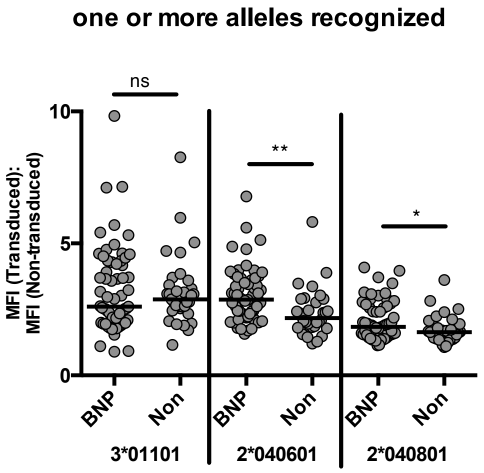

Reactivity to BoLA alleles was determined by flow cytometry and is compared between 63 BNP dam and 33 highly alloreactive, non-BNP dam sera that recognized one or more BoLA alleles. Grey circles symbolize individual sera, black bars the group median. One asterisk indicates a level of significance of $p<0.05$, two asterisks a level of $p<0.01$.
