# Supplementary material for: Bovine Neonatal Pancytopenia-Associated Alloantibodies Recognize Individual Bovine Leukocyte Antigen 1 Alleles
Source: Front Immunol. 2018 Aug 14;9:1902. doi: 10.3389/fimmu.2018.01902 (PMC6102493; doi:10.3389/fimmu.2018.01902)
Supplement: Supplementary file 2 [file data_sheet_2.pdf]

Supplementary Figure S2

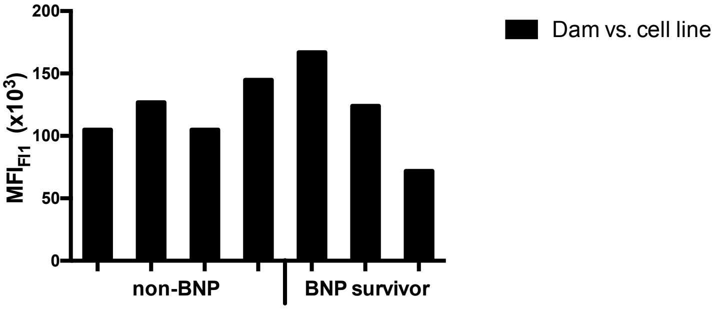

Serum reactivity to the production cell line was determined by flow cytometry for four Non-BNP and three BNP dams that were studied in Fig 7B.
